# A One Health approach for the genomic characterization of antibiotic-resistant *Campylobacter* isolates using Nanopore whole-genome sequencing

**DOI:** 10.3389/fmicb.2025.1540210

**Published:** 2025-01-29

**Authors:** Ana Hurtado, Medelin Ocejo, Beatriz Oporto, José Luis Lavín, Ruth Rodríguez, María Ángeles Marcos, Mikel Urrutikoetxea-Gutiérrez, Miriam Alkorta, José María Marimón

**Affiliations:** ^1^Animal Health Department, NEIKER – Basque Institute for Agricultural Research and Development, Basque Research and Technology Alliance (BRTA), Bizkaia Science and Technology Park 812L, Derio, Bizkaia, Spain; ^2^Applied Mathematics Department, NEIKER – Basque Institute for Agricultural Research and Development, Basque Research and Technology Alliance (BRTA), Bizkaia Science and Technology Park 812L, Derio, Bizkaia, Spain; ^3^Laboratorio de Salud Pública en Gipuzkoa, Donostia-San Sebastián, Gipuzkoa, Spain; ^4^Clinical Microbiology Service, Basurto University Hospital, Organización Sanitaria Integrada Bilbao-Basurto, Bilbao, Bizkaia, Spain; ^5^Biobizkaia Health Research Institute, Microbiology and Infection Control, Barakaldo, Bizkaia, Spain; ^6^Infectious Diseases Area, Microbiology Department, Biogipuzkoa Health Research Institute, Donostia-San Sebastián, Gipuzkoa, Spain; ^7^Donostialdea Integrated Health Organization, Donostia University Hospital, Donostia-San Sebastián, Gipuzkoa, Spain; ^8^Faculty of Medicine, University of the Basque Country, UPV/EHU, Donostia-San Sebastián, Gipuzkoa, Spain

**Keywords:** *Campylobacter jejuni*, *Campylobacter coli*, antimicrobial resistance, whole-genome sequencing, Nanopore long-fragment sequencing, one health, pangenome, resistome

## Abstract

In response to the growing threat posed by the spread of antimicrobial resistance in zoonotic *Campylobacter*, a One Health approach was used to examine the genomic diversity, phylogenomic relationships, and the distribution of genetic determinants of resistance (GDR) in *C. jejuni* and *C. coli* isolates from humans, animals (ruminants, swine, and chickens), and avian food products collected during a regionally (Basque Country, Spain) and temporally (mostly 2021–2022) restricted sampling. Eighty-three *C. jejuni* and seventy-one *C. coli* isolates, most exhibiting resistance to ciprofloxacin and/or erythromycin, were whole-genome sequenced using Oxford Nanopore Technologies long-fragment sequencing (ONT). Multilocus sequence typing (MLST) analysis identified a high genomic diversity among isolates. Phylogenomic analysis showed that clustering based on the core genome was aligned with MLST profiles, regardless of the sample source. In contrast, accessory genome content sometimes discriminated isolates within the same STs and occasionally differentiated isolates from different sources. The majority of the identified GDRs were present in isolates from different sources, and a good correlation was observed between GDR distribution and phenotypic susceptibility profiles (based on minimum inhibitory concentrations interpreted according to the EUCAST epidemiological cutoff values). Genotypic resistance profiles were independent of genotypes, indicating no apparent association between resistance and phylogenetic origin. This study demonstrates that ONT sequencing is a powerful tool for molecular surveillance of bacterial pathogens in the One Health framework.

## 1 Introduction

*Campylobacte*r spp. infections are among the most common causes of zoonotic foodborne bacterial diseases in the European Union (EU) and other countries (Kaakoush et al., 2015; EFSA and ECDC, 2024a). The most important human pathogens within the genus, *Campylobacter jejuni* and *Campylobacter coli*, colonize the intestinal tract of warm-blooded animals, including food-producing animals that can serve as sources of contamination of carcasses or milk. Human infection occurs by the consumption of contaminated food and water or by direct contact with animals or their feces (Newell et al., 2017). Many *Campylobacter* lineages associated with human infections are also recovered from multiple host species (host generalist lineages), while other genotypes are more commonly associated with specific reservoir species, such as poultry or ruminants (host specialist lineages) (Gripp et al., 2011; Noormohamed and Fakhr, 2014; Sheppard et al., 2014; Epping et al., 2021).

*Campylobacter* infections are generally mild but can be fatal among very young children, particularly in low- and middle-income countries (Black et al., 2024). The majority of infections do not require antibiotic treatment (Blaser and Engberg, 2008), but some patients may suffer more severe symptoms and require antibiotics. Ciprofloxacin or other fluoroquinolones were originally the recommended antibiotics for the treatment of severe human campylobacteriosis, but their efficacy has been compromised due to increasing levels of resistance (Yang et al., 2019; Inglis et al., 2021), which are high in *C. jejuni* and range from very high to extremely high in *C. coli* in the EU (EFSA and ECDC, 2024b). Macrolides such as azithromycin have now become the first-line treatment for severe infections as their resistance levels remain low, although resistance is slightly higher in *C. coli* than in *C. jejuni* (Shane et al., 2017; EFSA and ECDC, 2024b).

The increasing emergence of antibiotic-resistant campylobacters jeopardizes the effective treatment of campylobacteriosis in humans and poses a major public health challenge. The JIACRA IV report (2019–2021) found associations between antimicrobial resistance (AMR) in bacteria from humans and AMR in bacteria from food-producing animals for certain bacteria–antimicrobial combinations. Specifically, this was the case for the resistance to macrolides in *C. coli* from swine and humans (ECDC, EFSA, EMA, 2024).

Several studies have been carried out in the Basque Country (Spain) to monitor resistance in *C. jejuni* and *C. coli* in food-producing animals and to characterize the isolates from healthy livestock (Ocejo et al., 2019, 2021, 2023). Hospitals routinely analyze isolates from patients while public health laboratories monitor resistance in bacteria isolated from food products at the retail level. However, despite the complex circulation and interplay of strains between animals, food, and humans, the generated data had not been compared before. This study was designed to address the detection and epidemiological analysis of resistant *C. jejuni* and *C. coli* from a One Health perspective through a collaboration between healthcare, public health, and animal health sectors. For this, *Campylobacter* isolates from different sources (humans, food-producing animals, and food products), most of them resistant to ciprofloxacin and/or erythromycin, collected during a regionally (Basque Country, Spain) and temporally (mostly 2021–2022) restricted sampling, were whole-genome sequenced (WGS) using long-read Oxford Nanopore Technologies (ONT) with the following objectives: (i) examine genomic diversity, (ii) investigate the phylogenomic relationships among strains, and (iii) determine the distribution of genetic determinants of resistance (GDR). This study provides, for the first time, an overview of the genetic diversity of resistant *Campylobacter* isolates from different sources in the region, contributing to a deeper understanding of the epidemiology of *Campylobacter* infection.

## 2 Materials and methods

### 2.1 Isolation and identification of *Campylobacter* from animals, foodstuffs, and humans

The animal-derived isolates were provided by NEIKER (Derio, Bizkaia, Basque Country, Spain) and were obtained from rectal feces (ruminants and swine) or ceca (chicken) collected from 250 cattle, 41 sheep, 75 swine, and 95 chickens (mainly free-range) between 2021 and 2023 at 4 slaughterhouses in the Basque Country. For the isolation of thermophilic *Campylobacter* spp., samples from five animals (same animal species, slaughterhouse, and sampling date) were analyzed in a single 25 g pool, diluted 1:10 in Preston broth, homogenized, and incubated at 42°C for 18 ± 2 h for enrichment. Suspensions (0.1 mL) were then subcultured onto a Chromogenic-*Campylobacter* Selective Agar (CASA® Agar, bioMérieux) and incubated at 41 ± 1°C in a microaerobic atmosphere (5% O_2_, 10% CO_2_, 85% N_2_) for 48–72 h. Isolation from chicken cecal content was carried out by direct culture in CASA® agar. To confirm the presumptive *Campylobacter* isolates and identify the species present, individual colonies were tested using a multiplex real-time PCR targeting *C. jejuni mapA* gene and *C. coli ceuE* gene (Best et al., 2003).

*Campylobacter* isolates from food were provided by the *Laboratorio de Salud Pública en Gipuzkoa* (LSPG, Donostia-San Sebastián, Gipuzkoa, Basque Country, Spain) and had been isolated from food products in the retail trade in the Basque Country in 2019–2022. Food samples included chicken skin (210 samples analyzed in pools of three carcasses), various poultry meat products (sausages, hamburgers, and breast cuts; *n* = 405), and a spinach burger. Isolation and identification of *Campylobacter* spp. were carried out according to UNE-EN ISO 10272-1. The samples (10 g) were diluted 1:10 in Preston broth and homogenized. After incubation at 41 ± 1°C for 24 ± 2 h, a 10-μL loop was subcultured onto modified charcoal-cefoperazone-deoxycholate agar (mCCDA) plates or CampyFood agar (CFA, bioMérieux) and incubated at 41 ± 1°C in a microaerobic atmosphere for 44 ± 4 h. To confirm and identify the presumptive *Campylobacter* species, a commercial real-time PCR kit was used (SureTect™ *Campylobacter jejuni*, *C. coli* and *C. lari* PCR kit, Thermo Fisher Scientific).

Human isolates were collected in 2022 from patients with gastroenteritis in two different hospitals of the Basque Country Public Health Service (Osakidetza): *Hospital Universitario Basurto* (HUB) in Bilbao, Bizkaia and *Hospital Universitario Donostia* (HUD), in Donostia-San Sebastián, Gipuzkoa. No outbreaks of human infections were observed during the study period. Stool samples were screened for the presence of *Campylobacter* using a multiplex-PCR (BD MAX Enteric Bacterial Panel, Becton Dickinson, USA or Allplex, gastrointestinal panel Seegene, South Korea). Samples positive for *Campylobac*ter were cultured on *Campylobacter* agar (BBL, Becton Dickinson, Heidelberg, Germany) and incubated at 41 ± 1°C in a microaerobic atmosphere for 24–48 h. Colonies with morphology indicative of *Campylobacter* (small, mucoid, grayish colonies) were identified using MALDI-TOF (Bruker Daltonics, Bremen, Germany).

### 2.2 Antimicrobial susceptibility testing for WGS selection

To identify resistant *Campylobacter* isolates, antimicrobial susceptibility testing (AST) was performed. Isolates showing microbiological resistance to ciprofloxacin, erythromycin, or both were prioritized for WGS, with selection aimed at capturing the diversity of phenotypic resistance profiles among the different sources. For the selection of animal and food isolates, minimum inhibitory concentration (MIC) data were already available. For the selection of the human clinical isolates, preliminary AST was performed using the disk-diffusion method with ciprofloxacin (5 μg) and erythromycin (15 μg) disks following the European Committee for Antimicrobial Susceptibility Testing (EUCAST) guidelines and breakpoints.^1^ MICs were later determined for the isolates selected for WGS.

MICs were determined by broth microdilution using Sensititre® EUCAMP3 Susceptibility Plates (Thermo Fisher Scientific, Waltham, MA, USA) containing 2-fold serial dilutions of six antimicrobial agents: chloramphenicol (CHL), ciprofloxacin (CIP), ertapenem (ETP), erythromycin (ERY), gentamicin (GEN), and tetracycline (TET). Antimicrobials and serial dilution ranges were selected following recommendations by the Commission Implementing Decision 2020/1729/EU. The MIC results were interpreted using epidemiological cutoff (ECOFF) values as developed by the EUCAST to define microbiological resistance to the antimicrobial in question, that is, to discriminate those microorganisms with and without acquired resistance mechanisms (mutant and wild type, respectively). For ETP, with no defined ECOFF, a MIC_ETP_ > 0.5 mg/L was used as a reference based on EFSA recommendations (Amore et al., 2021). Multi-drug resistance (MDR) was defined as resistance to three or more antibiotic classes.

### 2.3 Long-read whole-genome sequencing (WGS) and bioinformatic analyses

Genomic DNA was extracted from single colony pure cultures using the NZY Microbial gDNA Isolation kit (NZYtech) for animal samples, and the QIAamp DNA kit (QIAGEN) for isolates from human and food origin. Libraries were prepared using the ONT rapid barcoding kit (SQK-RBK004) and run in FLO-MIN106 (R9.4.1) or FLO-MIN111 (R10.3) flow cells on a MinION Mk1C device (ONT). The output files generated by ONT sequencing were base-called in high accuracy mode (HAC) and quality-filtered using Guppy v6.4.6. Reads were adapter-trimmed with Porechop v.0.2.4 with the default parameters (Wick et al., 2017a) and filtered by length and quality using Filtlong v.0.2.0^2^ by discarding short reads (<1,000 bp). Then, the resulting fastq reads were *de novo* assembled using Unicycler (Wick et al., 2017b) or Flye (Kolmogorov et al., 2019). Raw sequences and assembly descriptive statistics were assessed with Seqkit stats v.2.5.1 (Shen et al., 2016). Assembled genome completeness and contamination levels were estimated using CheckM v.1.2.2 (Parks et al., 2015).

MLST profiles were determined from unassembled long reads using Krocus, a k-mer-based method for the rapid detection of multilocus sequence types (STs) from long-sequencing reads (database updated on 28/11/2023) (Page and Keane, 2018). When k-mer coverage is below 100%, the allele with the highest number of k-mers is identified, but with a low confidence flag. Genomes were processed to predict plasmid- and chromosome-derived contigs using PlasFlow (v.1.1) (Krawczyk et al., 2018). BLASTn v.2.12.0+ (Zhang et al., 2000) and ABRicate v.1.0.1^3^ were used to screen the genomes against VFDB_setB (full dataset) for virulence factors (VFs) (Liu et al., 2022) and against ResFinder v.2.2.1 (Zankari et al., 2012) for the detection of acquired antimicrobial resistance genes (ARGs). Additionally, a custom database containing two variants of the *aph(2″)-Ii* gene (accession numbers KX931104 and KX931110), described by Fabre et al., 2018 was created for complementary analysis with ABRicate. Searches were also performed against the NCBI database (Agarwala et al., 2016) to identify genes not covered in ResFinder (i.e., *aad9*, *satA*, *sat4*, and *spw*). Chromosomal point mutations associated with AMR were investigated by screening unassembled reads against the PointFinder database (Zankari et al., 2017) using Resfinder v.4.1.0 (Bortolaia et al., 2020). The databases used were the versions available on 24/11/2023. Resfinder hits were filtered at 90% coverage and identity. For virulence factors filtering thresholds were set at 90% coverage and 60% identity, and the pattern of presence/absence of these genes was used as a typing scheme for comparative genomic fingerprinting, supported with a dendrogram. The clustering method was performed using the unweighted pair-group method with arithmetic mean (UPGMA), based on the Jaccard distance matrix. The function hclust (v.3.6.1) of the R statistical package v.3.6.3 was used for the analysis.

To further explore the genomic diversity of each *Campylobacter* species, a pangenome analysis was performed on the European Galaxy platform.^4^ For this, genomes were first annotated with Prokka v.1.14.6 (Seemann, 2014), followed by the use of Roary v.3.13.0 (Page et al., 2015) to generate a matrix that maps the presence and absence of all genes, differentiating between core and accessory genes. Both tools were run with default parameters. Then, the presence/absence of accessory genes was used to construct a dendrogram through hierarchical clustering analysis as described above. To assess the phylogenetic relationship between isolates from different sources using the core genome structural and point variations (SNPs), Parsnp v1.7.4 (Treangen et al., 2014) along with the implemented RaxML v.8.2.12 (Stamatakis, 2014) was used with defaults parameters and specifying -r! parameter to randomly select the reference from the set of genomes analyzed. The resulting trees were visualized and annotated with iTOL (Letunic and Bork, 2007). A minimum spanning tree was constructed by the goeBURST algorithm using the Phyloviz v2.0 software (Nascimento et al., 2017) to visualize the relationships between the STs and the isolation source.

Plasmids harboring ARGs were compared by aligning their sequences using MAUVE in progressive mode (Darling et al., 2010) in Geneious Prime v.2020.2.4^5^ software and they were pairwise compared with FastANI v1.33^6^ to assess homology. In addition, they were compared with the approximately 60,000 complete plasmids contained in PLSDB (2023_11_03_v2) (Galata et al., 2019) using Mash, and the match with the lowest Mash distance was retained as the best hit.

### 2.4 Real-time TaqMan PCR targeting the C257T point mutation in the *gyrA* gene

Whenever a discrepancy was observed between phenotypic resistance to CIP and detection of the corresponding SNPs by WGS, real-time TaqMan PCR was used to detect the point mutation associated with resistance to quinolones (C257T in the *gyrA* gene, Thr-86-Ile) as described elsewhere for *C. jejuni* (Oporto et al., 2009) and *C. coli* (Ocejo et al., 2019).

### 2.5 Statistical analysis

Non-parametric tests and regression models were used to evaluate the distribution and presence of GDRs in *C. jejuni* and *C. coli* isolates and their sources. The Mann–Whitney *U*-test compared the total number of GDRs between species, while the Kruskal–Wallis test, followed by Dunn’s test with Bonferroni correction, assessed differences between sources within each species. To examine differences in the number of GDRs per AMR class between sources, Poisson regression models were applied separately for *C. coli* and *C. jejuni*. The total GDR count per AMR class served as the dependent variable, with the source as the explanatory variable.

For the comparison of the presence or absence of specific GDRs between sources, logistic regression models were used, treating each GDR as a binary outcome (presence/absence), and the source as the categorical predictor. Separate models were constructed for *C. coli* and *C. jejuni*. Odds ratios (ORs) and 95% confidence intervals (CIs) were calculated to estimate the likelihood of GDR presence in each source compared to a reference category. Pairwise comparisons were conducted only when the global model showed statistical significance. In all analyses, statistical significance was defined as a *p*-value of <0.05.

## 3 Results

### 3.1 Isolates included in the study and antimicrobial resistance profiles

Out of 1,637 *Campylobacter* isolates (1,363 *C. jejuni* and 274 *C. coli*) that underwent preliminary AST (Supplementary Table S1), a total of 154 isolates (83 *C. jejuni* and 71 *C. coli*) were selected for WGS. The selection comprised strains isolated in the Basque Country (northern Spain) between 2019 and 2023 (86.4% in 2021 and 2022) that exhibited microbiological resistance to CIP, ERY, or both, along with isolates representing a diversity of phenotypic resistance profiles and sources, i.e., human, animal (cattle, sheep, swine, and chicken) and food samples (mostly from chicken) (Table 1). All swine-derived isolates were identified as *C. coli*, whereas isolates from other sources included both *C. jejuni* and *C. coli*.

**Table 1:** Isolates included in the study.

Source	*n*	Type of sample	*C. jejuni*	*C. coli*	Total
**Human**			**32**	**22**	**54**
Human	54	Human stools	32	21	53
		Human blood	0	1	1
**Animal**			**32**	**35**	**67**
Chicken	23	Chicken ceca	12	11	23
Swine	16	Swine rectal feces	0	16	16
Ruminants	28	Bovine rectal feces	18	7	25
		Ovine rectal feces	2	1	3
**Foodstuff**			**19**	**14**	**33**
Chicken meat	18	Chicken skin	12	6	18
	2	Chicken breast	2	0	2
Processed poultry	12	Chicken burger	1	0	1
		Chicken sausage (whole meat)	1	3	4
		Chicken sausage (minced meat)	1	4	5
		Spicy chicken mince	1	0	1
		Turkey sausage (whole meat)	0	1	1
Processed vegetable	1	Spinach burger	1	0	1
**Total**			**83**	**71**	**154**

Words and values in bold represent the subtotal for each main source category of isolates.

As a consequence of the selection criteria, the majority of the 154 isolates included in this study were resistant to at least one of the antimicrobials tested (*n* = 130), 11 could not be tested by broth microdilution but were resistant to either CIP or ERY by disk-diffusion, and 13 were fully susceptible. The complete list of all isolates, including available metadata such as sample source and sampling date, is provided in Supplementary Table S2. Among the selected isolates tested by broth microdilution (*n* = 143), a high percentage were resistant to CIP (81.8%; 58 *C. coli* and 59 *C. jejuni*) and TET (76.2%; 61 *C. coli* and 48 *C. jejuni*) recovered from all sources (livestock, foodstuffs, and humans). In 14.7% of the tested isolates (6 *C. jejuni* and 21 *C. coli*), MIC values for ETP were above the reference value (MIC_ETP_ > 0.5 mg/L), but only five *C. jejuni* isolates from humans and foodstuffs showed an ETP MIC >2 mg/L. All *C. jejuni* isolates were susceptible to ERY but 23 *C. coli* (from all sources) were ERY-resistant, all in combination with resistance to CIP. Resistance to GEN was only detected in eight *C. coli*. Overall, MDR was more widespread in *C. coli* than in *C. jejuni*. Hence, MDR in *C. jejuni* was restricted to five food isolates resistant to three antimicrobial classes, whereas *C. coli* MDR isolates were recovered from the three sources and included isolates resistant to three (*n* = 25), four (*n* = 6), and five (*n* = 2) classes.

### 3.2 WGS output and assembly results

ONT sequencing provided a median of 24,522 reads per sample [interquartile range (IQR) = 12,789–34,764] at a median of 169 Mb per sample (IQR = 66.1–240.5 Mb), corresponding to 99× median of coverage value (IQR = 39–141×). The N50 median value was 9,548 bases per sample (IQR = 6,663–12,250). The assembled genomes exhibited a median completeness of 89.3% (IQR = 83.1–93.2%) and a contamination level of 0.5% (IQR = 0.3–0.8%). Information regarding sequencing output and assembly statistics is provided in Supplementary Table S3.

### 3.3 Genomic diversity characterization

**Figure 1:**
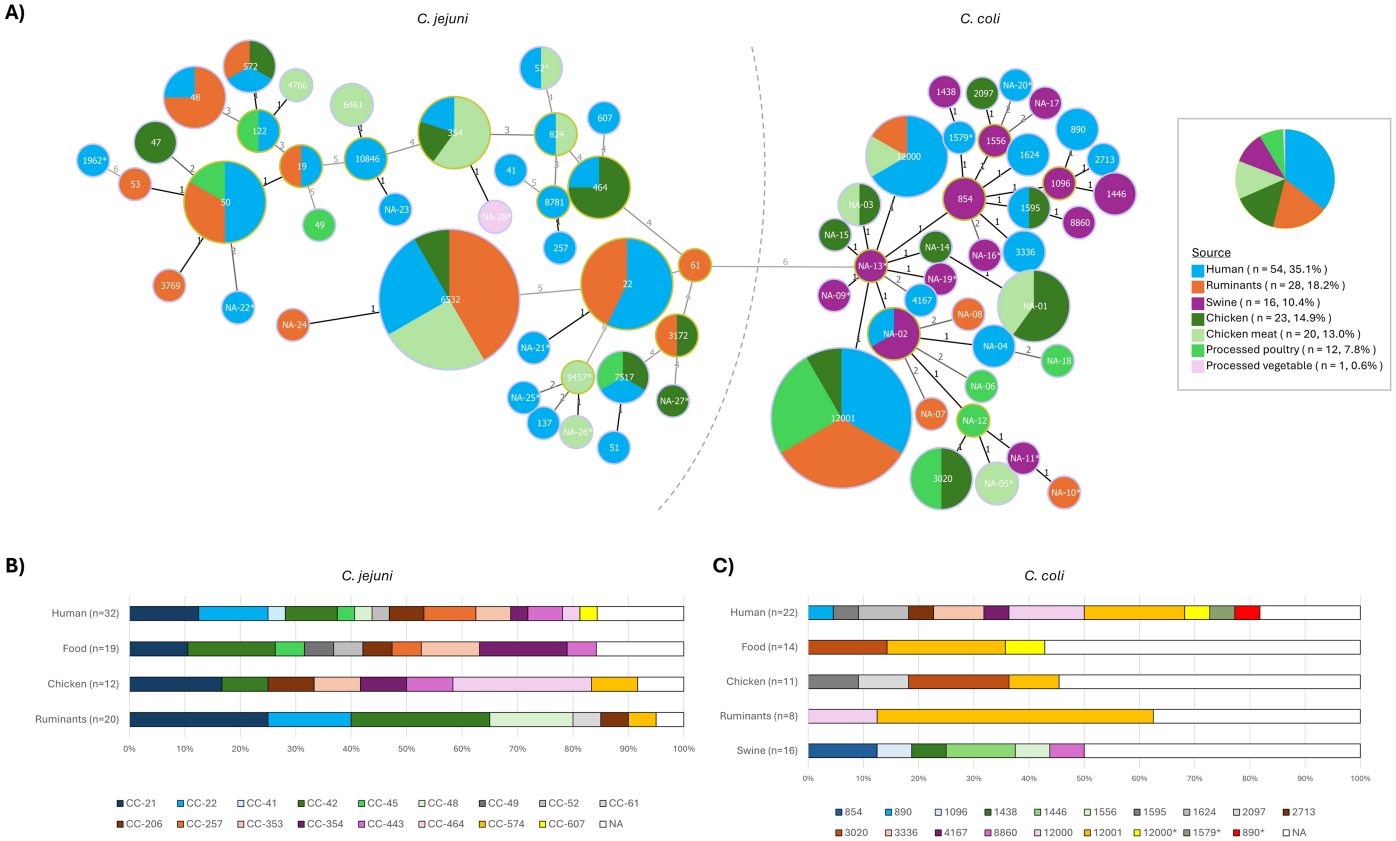
MLST profiles distribution of *C. jejuni* and *C. coli* isolates in the different isolation sources. **(A)** Minimum spanning tree where each node represents a sequence type (ST) and its size correlates with the number of isolates. A separate pie chart is provided to illustrate the proportion of isolates by source. **(B)** Stacked bar plot represents the distribution of clonal complexes (CCs) in *C. jejuni*. **(C)** Stacked bar plot represents the distribution of STs in *C. coli*. NA, not assigned ST; asterisks (NA-nr*) are used to refer to STs with low confidence assignment.

The MLST results are presented in Supplementary Table S4, and a minimum spanning tree showing the relatedness among STs and their distribution according to the isolation source is depicted in Figure 1.

Of the 83 *C. jejuni* genomes, 75 were assigned an MLST sequence type (ST; 7 of them with a certain degree of uncertainty - ST*) and 8 remained non-assigned due to unreliable allele calls (*n* = 6, NA*) or potentially novel profiles resulting from a novel combination of alleles (*n* = 2, NA) (Supplementary Table S4). The 30 assigned STs belonged to 17 clonal complexes (CCs). The most abundant *C. jejuni* type was ST-6532 (*n* = 12, CC-42), isolated from humans (*n* = 3), cattle (*n* = 5), chicken cecum (*n* = 1), and chicken meat (*n* = 3). Other less prevalent ST types recovered from the three sources (humans, animals, and foodstuffs) were ST-50 (*n* = 6) and ST-354 (*n* = 5) (Figure 1). Six STs were shared by *C. jejuni* isolates recovered from humans and ruminants, and nine from humans and poultry-associated isolates (animals and/or food).

Of the 71 *C. coli*, 42 genomes were assigned to 17 STs (4 of them with a certain degree of uncertainty - ST*) and 29 remained non-assigned due to unreliable allele calls (*n* = 11, NA*) or potentially novel types (*n* = 18, NA). All assigned STs belonged to clonal complex CC-828. The most abundant *C. coli* type was ST-12001 (*n* = 12), isolated from humans (*n* = 4), cattle (*n* = 4), chicken meat (*n* = 3), and chicken cecum (*n* = 1) (Figure 1). Two other STs were associated with isolates from humans and animals, i.e., ST-12000 (humans, cattle, and chicken meat) and ST-1595 (human and chicken).

**Figure 2:**
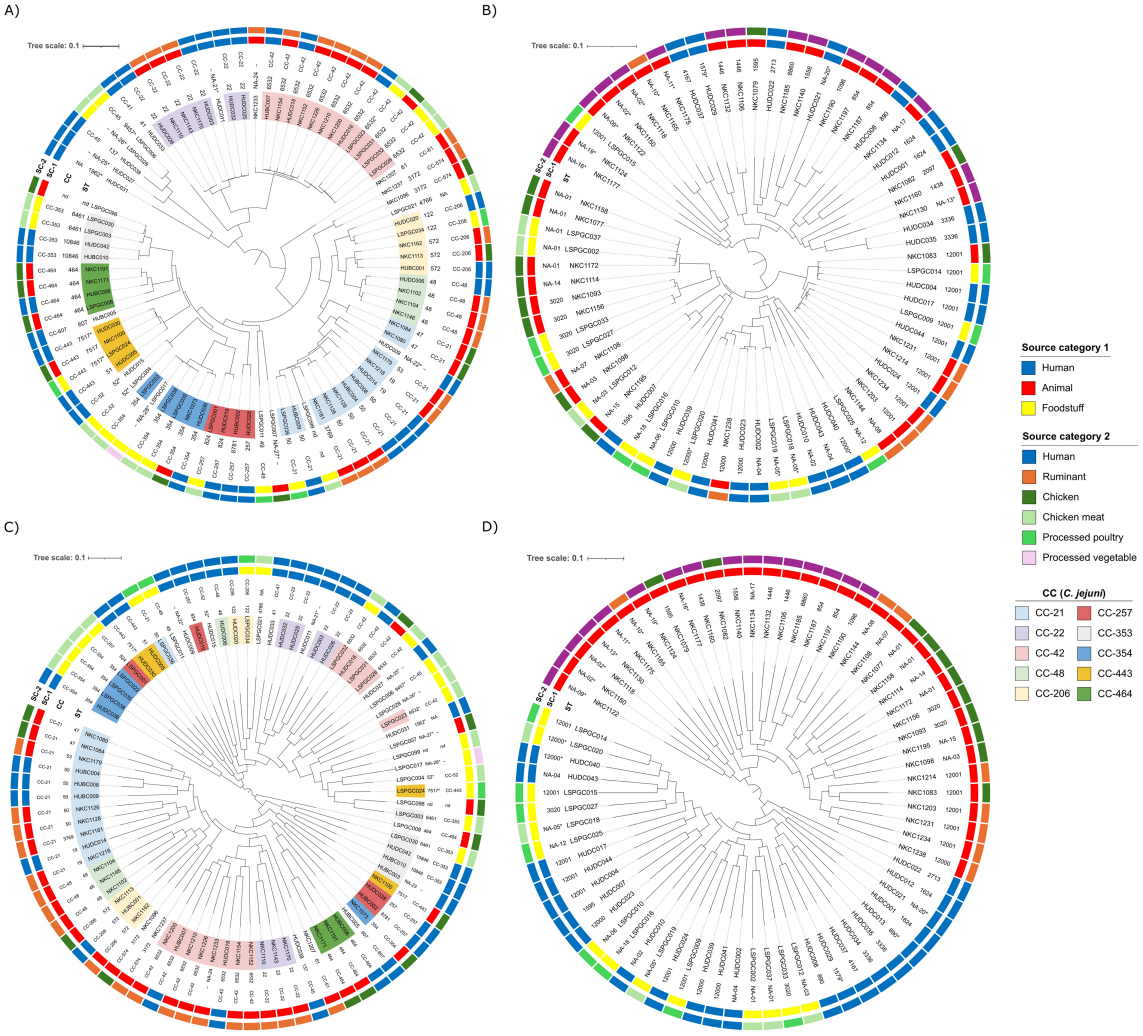
Pangenome analysis of *Campylobacter* isolates from different sources. SNP-based core genome phylogeny of *C. jejuni* **(A)** and *C. coli* **(B)** isolates generated by Parsnp and RAxML, illustrating genetic relatedness between isolates. Dendrogram represents the hierarchical clustering of the genomes based on the presence/absence of accessory genes in *C. jejuni* **(C)** and *C. coli* **(D)** genomes. Isolation source (source category SC-1 – inner ring and source category SC-2 – outer ring) and MLST (ST and CCs) are provided for each isolate. *C. jejuni* isolates belong to CCs represented by more than three isolates are color-coded according to legend **(A,C)**; all assigned STs of *C. coli* belong to the CC-828 **(B,D)**. nd, not done; NA, not assigned; asterisks (NA-nr*) are used to refer to STs with low confidence assignment.

To investigate the genomic relatedness among the strains, pangenome analysis was performed for each *Campylobacter* species separately. After excluding two human isolates (one of each species) from the analysis (MUMi >0.01), Parsnp produced alignments of the core genes that covered 56% of the average *C. jejuni* genome size, and 57% of the average *C. coli* genome size. The SNP-based phylogenetic trees inferred from these core genome alignments showed that *C. jejuni* isolates of the same ST and CCs clustered together (Figure 2A and Supplementary Figure S1). Although with few exceptions, a similar association was also observed for *C. coli* (Figure 2B and Supplementary Figure S2); the exceptions were the two ST-1595 isolates of chicken and human origin that clustered distantly apart from each other and one of the 12 ST-12001 isolates that diverged from the rest. The dendrograms built based on the presence/absence of accessory genes included two large clusters in both species. In *C. jejuni*, 1 cluster included isolates from humans (*n* = 17), chicken (16 food products and 2 ceca), and the spinach burger (*n* = 1), and the other cluster comprised all the ruminant isolates (*n* = 20), 15 human isolates, and 10 isolates from chicken (1 food product and 9 ceca) (Figure 2C and Supplementary Figure S3). The analysis of the accessory genome of *C. coli* resulted in two large clusters, separating all the animal isolates (*n* = 35) from the human and foodstuff isolates (*n* = 36) (Figure 2D and Supplementary Figure S4). Accessory genome-based associations were sometimes independent of the MLST typing results in both species, thus separating isolates that grouped together in the core genome-based parsnp tree into distinct clusters.

When the genomes were analyzed for the presence/absence of genes coding for VFs using the full dataset of the VFDB, a total of 261 VF hits were obtained with different levels of identity (Supplementary Figure S5). Ten *C. jejuni* genomes (8 CC-21, 1 NA-22*, and 1 nd) were the only ones carrying genes responsible for the synthesis and modification of lipooligosaccharides (LOS) such as Cj1135, Cj1136, Cj1137c, Cj1138, *cstIII*, *neuA1*, *neuB1*, *neuC1*, and *wlaN*. In general, isolates within the same CCs clustered together based on the VF profile; the main exception was CC-21, which split into three different clusters, one of them including the genomes harboring the abovementioned LOS genes.

### 3.4 Detection of genes and chromosomal point mutations associated with antimicrobial resistance

**Figure 3:**
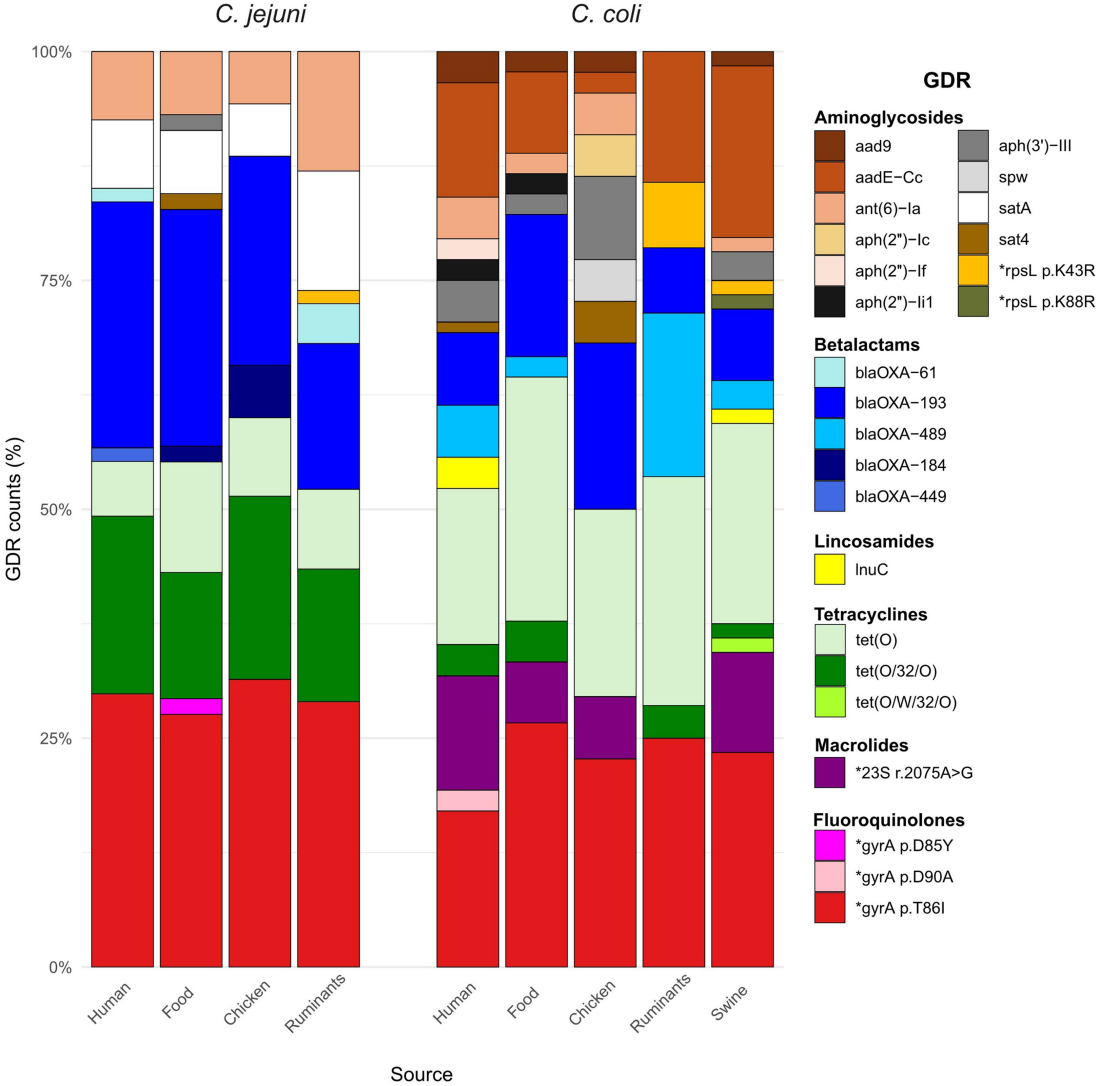
Stacked bar plots representing the distribution of genetic determinants of resistance (GDRs) in the different sources for each *Campylobacter* species. GDRs are color-coded according to the legend and organized by their corresponding antimicrobial class.

A total of 19 acquired ARGs and 6 single nucleotide point (SNP) mutations in 3 other genes (*gyrA*, 23S rRNA, *rpsL*) were identified (Figure 3 and Supplementary Figures S6, S7). GDRs were more widespread in *C. coli* than in *C. jejuni*; 8 of the 25 GDRs detected were present in both *Campylobacter* species, 12 were only detected in *C. coli*, and 5 only in *C. jejuni*. However, no significant differences in the total number of GDRs per isolate were found between *C. coli* (median = 4) and *C. jejuni* (median = 3) resistant isolates (W_Mann–Whitney_ = 2032.5, *p* = 0.06) or in the median total GDRs per isolate across the different sources. Detected GDRs were associated with resistance to six different classes of antimicrobials, and their distribution varied between *C. jejuni* and *C. coli* but were similar in all sources (Figure 3). No significant differences were observed in the presence of any GDR between sources, except for the gene *aadE-Cc*, where chicken had the lowest prevalence. However, the difference was only significant when compared to swine, and with a very small odds ratio (OR = 0.03, *p* = 0.036).

Resistance to quinolones was coded by three chromosomal point mutations in the *gyrA* gene. The T86I GyrA mutation was the most prevalent (*n* = 126), D90A was found in two human *C. coli* that also carried the T86I mutation, and one *C. jejuni* isolated from chicken meat carried the D85Y mutation. Twenty-four *C. coli* carried a point mutation associated with erythromycin resistance (A2075G) in the 23S rRNA gene, which was not present in susceptible isolates.

GDRs coding for resistance to different aminoglycosides included genes associated with resistance to streptomycin that were present in isolates recovered from all sources such as *aadE-Cc* (32 *C. coli*) and *ant(6)-Ia* (20 *C. jejuni* and 8 *C. coli*), and two-point mutations in *rpsL* only found in isolates from animals (p.K43R in 3 *C. coli* and 1 *C. jejuni* and p.K88R in 1 *C. coli*). The gene that mediates resistance to amikacin *aph(3′)-III*, albeit less common, was also present in isolates from all sources (1 *C. jejuni* and 11 *C. coli*). The genes encoding gentamicin resistance were sporadically found and were restricted to *C. coli.* Three variants of *aph(2″)* were identified: *aph(2″)-Ic* (2 chicken ceca), *aph(2″)-If* (2 human), and *aph(2″)-Ii_1_* (2 human and 1 chicken sausage). Other genes associated with aminoglycoside resistance found were *aad9* (6 *C. coli*) and *spw* (2 *C. coli*).

Tetracycline resistance genes found were *tet*(O), *tet*(O/32/O), and *tet*(O/W/32/O), with *tet*(O) being more prevalent in *C. coli* and *tet*(O/32/O) in *C. jejuni* regardless the isolation source; *tet*(O/W/32/O) was only detected in one *C. coli* isolated from swine. Regarding *β*-lactams, five different *bla*_OXA_ genes were identified in 113 isolates. Specifically, genes coding for oxacillinases of the OXA-184-like family were sporadically detected and only in *C. jejuni*, i.e., 5 isolates (2 human, 1 chicken burger and 2 chicken ceca) carried the *bla*_OXA-184_ gene and one human isolate carried the *bla*_OXA-449_ gene; gene alleles of the *bla*OXA-61-like family were more widespread and included *bla*_OXA-193_ (61 *C. jejuni* and 29 *C. coli*), *bla*_OXA-489_ (13 *C. coli*), and *bla*_OXA-61_ (4 *C. jejuni*). The *lnu(C)* gene, which confers resistance to lincomycin, was found in four *C. coli* (three humans and one swine) isolates. Two genes of the streptothricin acetyltransferase gene family, *satA* and *sat4*, were found in twenty and four isolates, respectively.

Although the majority of ARGs were located in the chromosome, six plasmids harbored ARGs. Thus, four *C. jejuni* isolates (two cattle, one chicken skin, and one chicken sausage) carried the *tet*(O) gene in plasmids ranging in size from 41.6 to 46.8 kbp; and two human *C. coli* carried the *tet*(O) – *aph(2″)-Ii_1_* – *aph(3′)-III* genes in plasmids of *ca.* 48 kbp. ProgressiveMAUVE multiple alignments showed that plasmids hosted by each *Campylobacter* species shared similar structures with a high degree of synteny (Supplementary Figure S8). The fastANI results showed 99.5% homology between both *C. coli* plasmids and 92.5–97.4% pairwise homology between the four *C. jejuni* plasmids. However, the two avian *C. jejuni* plasmids were more similar to each other (97.4%) and to the two human *C. coli* plasmids (97.1 and 99.1%) than to the two bovine *C. jejuni* plasmids (<95%) (Supplementary Table S5A). A Mash analysis of these plasmids revealed a high degree of homology with several *Campylobacter* plasmids from the PLSDB database (Supplementary Table S5B), originating from both human and chicken sources.

### 3.5 Comparison of phenotype (MIC) and genotype (WGS) data: correlation between susceptibility phenotypes and genotypes

Phenotypic (broth microdilution MIC-based) and genotypic (WGS-based) susceptibility results were compared. Resistant WGS genotypes were defined by the presence of one or more resistance genes and/or point mutation for each of the antimicrobials tested by broth microdilution. Discrepancies were observed in 14 genomes and were associated with susceptibility to CIP (6 *C. jejuni* and 2 *C. coli*), TET (3 *C. jejuni*), and GEN (6 *C. coli*) (Supplementary Figures S6, S7). Discrepancies between phenotypic CIP susceptibility and SNP detection by WGS were primarily associated with low genome coverage (<25X) and completeness (<85%). These discrepancies were resolved using the real-time TaqMan PCR assays, which detect the quinolone resistance-associated C257T point mutation in the *gyr*A gene (Thr-86-Ile). Discrepancies in two GEN-resistant *C. coli* isolates were resolved by including the *aph(2″)-Ii*_1_ gene variant (KX931104) in the analysis, which was not included in the default ResFinder database.

## 4 Discussion

This study provides a comprehensive genomic analysis of *C. jejuni* and *C. coli* isolates (the majority of which are resistant) within a One Health framework. It examines samples from humans (clinical isolates), animals (cattle, sheep, swine, and chicken), and food products (mainly chicken) from the same region (Basque Country, Spain), with most samples collected over a 2-year period (2021–2022). Long-read WGS was used to evaluate genomic diversity, examine the distribution of genetic determinants of resistance, and investigate the genomic relatedness among the strains from different sources.

Long-read sequencing offers significant advantages over short-read sequencing for genome assembly, often resulting in closed chromosomes and plasmids, which is advantageous for analyses based on the presence/absence of genes (ARGs, VFs, or accessory genome content). Conversely, long-read ONT sequencing has limitations in SNP-calling compared to short-read sequencing technologies such as Illumina, due to its higher inherent error rate (Delahaye and Nicolas, 2021). Correct allele assignment is essential for reliable MLST typing and the detection of SNPs associated with AMR. In this study, MLST was performed using Krocus, a k-mer-based bioinformatic tool specifically designed for MLST inference from unassembled long reads. While Krocus effectively classified the majority of isolates, several genomes could not be assigned to STs due to unreliable allele calls or novel combinations of alleles. Although this tool is not recommended for the detection of new alleles or the description of novel allele combinations (Page and Keane, 2018), tentative allele assignment still proved valuable for inferring relatedness between isolates. Notably, the majority of genomes with unreliable allele calls (17/27) had a sequencing coverage below 35x (mean: 19x), indicating that higher coverage improves the reliability of allele assignment by providing a greater number of reads. Furthermore, incorrect base calls associated with strand-specific methylation may have affected typing results as reported elsewhere (Dabernig-Heinz et al., 2024). Improvements in ONT sequencing quality, including Q20+ chemistry and super-accurate basecalling model, have been shown to increase raw read accuracy and are expected to improve SNP-calling reliability (Wagner et al., 2023).

The MLST revealed a wide genomic diversity in both *C. jejuni* and *C. coli* and phylogenomic analysis showed that core genome clustering of isolates from different sources was largely consistent with MLST profiles. However, accessory genome content occasionally discriminated isolates within the same STs based on source, sometimes also supported by VF-based clustering, highlighting the role of the accessory genome in niche adaptation and source differentiation (Epping et al., 2021). The *Campylobacter* isolates investigated in this study displayed a considerable ST diversity, including both widely distributed and rarely reported types. For example, the most abundant *C. jejuni* STs in this study, detected in humans, cattle, chicken, and chicken meat, was ST-6532, and it was the only ST of the ruminant-specialist complex CC-42. ST-6532 is not frequently reported, with only 30 records (0.02%, all human isolates) in the PubMLST database (135,571 isolates, data accessed 9 July 2024). However, it appears to be common in Spain, having been sporadically detected in sheep and cattle in the Basque Country, northern Spain (Ocejo et al., 2021, 2023), and identified as one of the most common STs among *C. jejuni* clinical isolates in a tertiary hospital in southern Spain (Fernández-Palacios et al., 2024). Interestingly, ST-6532 isolates clustered together in the SNP-based phylogenetic tree, but in the accessory genes’ presence/absence dendrogram, they separated into two distant clusters—one formed by chicken and the other by bovine isolates. Human isolates appeared in both clusters, a pattern that was also observed in the VF dendrogram. The second most prevalent ST identified in this study was ST-22, which belongs to CC-22. Recently classified as a cattle-specific CC (Epping et al., 2021), CC-22 is commonly associated with patients experiencing post-infectious complications of campylobacteriosis, such as Guillain–Barré syndrome and irritable bowel syndrome (Nielsen et al., 2010; Peters et al., 2021). In this study, ST-22 was the sole representative of CC-22, with isolates clustering together based on their core genome and sharing a similar VF profile while showing clear differences in accessory genome content between ruminant and human isolates.

Generalist CCs such as CC-21 and CC-206 were also detected in this study, with *C. jejuni* isolates recovered from humans sharing STs with those from ruminant and poultry sources. CC-21, one of the most widespread clonal complexes and common cause of human infection (Sheppard et al., 2009; Jolley et al., 2018), was recovered here from humans, chickens, sheep, and cattle. These isolates clustered together in both core and accessory gene analyses despite representing six different STs, with the exception of the two food isolates, which clustered separately in the accessory genome. Their GDRs and VFs were, however, diverse. Eight of the twelve CC-21 isolates (from humans, animals, and food) carried LOS biosynthesis locus genes, which are associated with Guillain–Barré syndrome, a finding consistent with previous reports on CC-21 isolates (Habib et al., 2009; Ocejo et al., 2023). Similarly, CC-206 isolates, which included two STs, consistently clustered according to their STs in all types of analyses, regardless of their source. In contrast, isolates in the generalist CC-48, recovered from humans and cattle, clustered together in the core genome phylogeny and shared a similar VF profile, but were separated by source on the accessory genome analysis. Poultry-specialist CCs (CC-257, CC-353, CC-354, and CC-464) were only represented by isolates from poultry, poultry products, and humans but none from ruminants. Based on the core genome analysis, these poultry-related CCs were more closely related among themselves than to cattle-related lineages. In addition, poultry-specific clonal complexes showed a more diverse population structure in the core genome than ruminant-associated lineages (CC-42 and CC-22), as reported before (Epping et al., 2021). The more recent onset of *C. jejuni* colonization of cattle compared to poultry has been proposed as an explanation for the closer clonal structure observed in cattle-associated lineages than in poultry-associated lineages (Sheppard et al., 2018; Mourkas et al., 2020).

In *C. coli*, ST diversity was larger, with only three STs represented by more than three isolates (ST-12000, ST-12001, and NA-01). Three STs (ST-12000, ST-12001, and ST-1595) were shared between isolates from humans and other sources. Types ST-12000 and ST-12001, first described in cattle from the Basque Country (Ocejo et al., 2023), were recovered here from humans and cattle (ST-12000), and from humans, cattle, chicken ceca, and processed chicken meat (ST-12001). Isolates within each of these STs clustered together in the core genome-based tree, but human isolates separated from ruminant isolates, clustered instead with isolates from poultry food products in the accessory genome dendrogram. Interestingly, *C. coli* isolates from swine belonged to STs not identified among the human isolates, and although they clustered with several human isolates in the parsnp tree, their accessory genome was clearly different. Notably, accessory genome analysis revealed source-specific clustering in some cases, especially in *C. coli*, highlighting a potential differentiation in accessory gene content driven by source-specific adaptation. For example, based on the accessory genome content, *C. coli* isolates recovered from humans and food products were separated from animal-derived isolates, with swine isolates clustering together. These findings may also suggest that *C. coli* infections in humans within the study region are more likely linked to poultry-derived foodstuffs rather than swine sources from the sampled slaughterhouses. Although less pronounced, a similar tendency was observed in the accessory genome dendrogram for *C. jejuni*. Human isolates were mainly related to food isolates, but they were also interspersed with isolates from ruminant sources, suggesting an important role of ruminants in *C. jejuni* human infections in the Basque Country. However, the potential role of animal-derived products cannot be entirely excluded, as human consumption of animal products is not restricted to those slaughtered in the region. On the other hand, unlike poultry food products, the majority of chicken ceca were collected from free-range chicken. Further investigations, including broader sampling and tracing of food supply chains, would be needed to clarify this hypothesis. An in-depth analysis of *C. coli* genomes would also be needed to investigate whether swine-specific *C. coli* lineages carry a specific pool of allelic variants of the core genome and accessory genes as reported for *C. jejuni* (Epping et al., 2021). These results suggest that accessory genome content might contribute to niche adaptation within the *Campylobacter* population, even among isolates sharing similar core genomes.

Since resistance to CIP, ERY, or both was one of the main criteria for the selection of isolates for this study, the widespread presence of GDRs was expected and as reported elsewhere (Ocejo et al., 2021, 2023), their presence generally correlated well with their phenotypic susceptibility profiles. Macrolide resistance to ERY was exclusively associated with the A2075G point mutation in the 23S rRNA gene. Notably, the *erm(B)* gene, which encodes an rRNA adenine N-6-methyltransferase that modifies the target binding site for macrolides in the 23S rRNA (Payot et al., 2006), was never detected. Discrepancies associated with CIP resistance were attributed to low sequencing quality and were all resolved using real-time TaqMan PCR assays targeting the C257T point mutation in the *gyrA* gene. Similarly, poor sequencing quality could have also contributed to the failure to detect any gene encoding TET resistance in two *C. jejuni* isolates that phenotypically expressed resistance. Conversely, the susceptibility to tetracyclines observed in a *C. jejuni* isolate carrying the *tet*(O/32/O) gene may suggest that it is a silent resistance gene. Silent resistance genes are functional genes that are not expressed and therefore do not confer phenotypic resistance, which, although silent in the host strain, can be activated and confer resistance when transferred to another bacterial strain (Deekshit and Srikumar, 2022). Finally, despite good sequencing quality, the genetic determinant responsible for gentamicin resistance could not be identified in three *C. coli* isolates (MIC_GEN_ = 16 mg/L), a finding already reported in other studies (Fabre et al., 2018; Zarske et al., 2024). High-level gentamicin resistance in *Campylobacter* is mainly associated with the presence of *aph(2″)* genes, for which several variants have been described (Toth et al., 2009; Smith et al., 2019). In this study, the additional screening for an *aph(2″)* gene variant, *aph(2″)-Ii*_1_ (Fabre et al., 2018), resolved discrepancies for two GEN-resistant *C. coli* isolates, as this variant was not included in the default ResFinder database. However, the inability to identify a GDR for the remaining isolates suggests the existence of another, as yet undescribed mechanism of gentamicin resistance.

The genetic profiles of AMR were independent of the MLST types, indicating no apparent association between AMR patterns and phylogenetic origin, as reported elsewhere (Ocejo et al., 2021, 2023; Zarske et al., 2024). This may be indicative of frequent horizontal and dynamic gene transfer, where ARGs are exchanged between strains of different phylogenetic origin, often driven by selective factors rather than by a lineage-specific relationship. As expected, GDRs were more widespread in *C. coli* than in *C. jejuni* (Cobo-Díaz et al., 2021; Ocejo et al., 2021, 2023; Zarske et al., 2024), reflecting broader genomic plasticity and capacity for resistance gene acquisition in *C. coli*. While some GDRs were shared between the two species, others were only found in each *Campylobacter* species. Albeit not significantly, GDRs encoding aminoglycoside resistance were more abundant, both in number and variety, in *C. coli* than in *C. jejuni*. In contrast, *bla*_OXA_ genes were more widespread (in number and type) in *C. jejuni* compared to *C. coli*. The only GDRs found in human isolates but not in isolates recovered from other sources were *bla*_*OXA*-449_, *aph(2″)-If*, and *gyrA* p.D90A, predominantly in *C. coli*.

Although detected in a small number of cases, the presence of *lnu(C)* gene, which codes for a lincosamide nucleotidyltransferase, in *C. coli* from humans and swine is remarkable since resistance to lincosamides is rarely reported in *Campylobacter*. The *lnu(C)* gene was first detected in *Streptococcus agalactiae* (Achard et al., 2005), and its presence in *Campylobacter* was first reported in two human *C. coli* isolates in the USA in 2016 (Zhao et al., 2016). However, a recent analysis of all the *lnu*(C)-harboring *Campylobacter* genomes available in GenBank up to May 2022 revealed its presence in 391 genetically diverse *C. coli* isolated from swine, chicken, humans, and food in 8 countries (Li et al., 2023). These findings suggest that while rare, the *lnu(C)* gene is globally distributed and linked to diverse hosts and environments. The infrequent reporting of lincosamide resistance in *Campylobacter* may largely be due to the fact that it is not routinely considered in standard antimicrobial susceptibility testing. Thus, WGS could serve as a useful tool to infer the prevalence of resistance to this class of antibiotics by identifying associated GDRs.

The AMR genes were mainly located on the chromosome and were only rarely found on plasmids. The six ARG-encoding plasmids identified carried the *tet*(O) gene, alone (four *C. jejuni* isolates of cattle and food) or together with the aminoglycoside resistance genes *aph(2″)-Ii*_1_ and *aph(3′)-III* (two human *C. coli*). The plasmids shared a common plasmid backbone, being quite similar among themselves particularly if hosted by the same *Campylobacter* species. However, the fact that the two human *C. coli* plasmids were more similar to *C. jejuni* plasmids from chickens, observed both in this study and the PLSDB database, suggests that they might have originated from a pTet plasmid ancestor of avian origin, with subsequent insertion of the aminoglycoside resistance gene cluster. In any case, natural transformation mediated by homologous recombination is the dominant mechanism for gene exchange in *Campylobacter* spp. (Guernier-Cambert et al., 2021).

In conclusion, this study provided an overview of the genetic diversity of resistant *C. jejuni* and *C. coli* strains isolated from humans, animals, and food in the same region during the same time period, with the aim of linking human infections to potential source types. The pangenome analysis showed that the majority of human *C. jejuni* isolates were closely related to those from food and/or animal (chicken and ruminant) sources. Exceptionally, in CC-22 and CC-48, the accessory genome of human isolates was more similar to poultry isolates than to ruminant isolates. In *C. coli*, human isolates were more closely related to isolates from chicken and poultry food products than to those from ruminants and swine. These findings suggest that within the studied population, poultry sources may pose a higher risk for human *C. coli* infections than ruminants or swine, while for *C. jejuni* the risk might be genotype-dependent. The majority of the GDRs were shared between isolates from different sources, and the resistance genetic profiles were independent of genotypes, indicating no apparent association between resistance and phylogenetic origin. Our study reinforces that ONT sequencing is a powerful tool for molecular characterization and surveillance of bacterial pathogens in the framework of a One Health approach.

## Data Availability

Raw sequencing data of the strains analyzed in this study are available at the NCBI Sequence Read Archive (SRA) database under accession numbers as detailed in Table S2, associated with the BioProject PRJNA1193326.

## Footnotes

^1^ https://www.eucast.org/

^2^ https://github.com/rrwick/Filtlong

^3^ https://github.com/tseemann/abricate

^4^ www.usegalaxy.eu

^5^ https://www.geneious.com

^6^ https://github.com/ParBLiSS/FastANI
